# Adjunctive Cerebrolysin therapy following mechanical thrombectomy for tandem right internal carotid artery and middle cerebral artery occlusion with residual post-procedural perfusion deficit: a case report

**DOI:** 10.25122/jml-2026-0102

**Published:** 2026-08

**Authors:** Roni Eichel, Stefan Mausbach, Michael Teicher, Natan Meir Bornstein

**Affiliations:** 1 Department of Neurology, Shaare Zedek Medical Center, Faculty of Medicine, Hebrew University of Jerusalem, Jerusalem, Israel

**Keywords:** adjunctive therapy, Cerebrolysin, cerebroprotection, anterior circulation stroke, tandem internal carotid artery occlusion, post-thrombectomy CT perfusion, wake-up stroke

## Abstract

Acute ischemic stroke patients may demonstrate persistent tissue-level hypoperfusion despite successful angiographic recanalization after mechanical thrombectomy. We report a 57-year-old man with wake-up stroke due to tandem occlusion of the right internal carotid artery and M1 middle cerebral artery who presented with left hemiparesis, neglect, and dysarthria (National Institutes of Health Stroke Scale, NIHSS, score 7). Computed tomography perfusion showed salvageable penumbral tissue, and he underwent mechanical thrombectomy with carotid stenting, achieving complete angiographic recanalization (Thrombolysis in Cerebral Infarction grade 3); no intravenous thrombolysis was given. However, post-procedural CT perfusion revealed persistent residual hypoperfusion despite macrovascular reperfusion. Adjunctive Cerebrolysin therapy was initiated immediately after the procedure. The patient demonstrated rapid neurological improvement (NIHSS 1 within hours), without treatment-related adverse effects, and was discharged on hospital day 5 with mild residual cognitive deficits. This case highlights the dissociation between angiographic and tissue-level reperfusion after thrombectomy and supports further investigation of adjunctive cerebroprotective strategies guided by post-procedural perfusion imaging. Registered at ClinicalTrials.gov (NCT06070753).

## Introduction

Endovascular thrombectomy (EVT) has transformed the management of acute ischemic stroke due to large vessel occlusion, with the HERMES collaboration demonstrating successful angiographic recanalization in approximately 71% of treated patients. [1] Despite this success, approximately half of all successfully recanalized patients fail to achieve functional independence at 90 days — a phenomenon termed “futile recanalization” [2–4].

Multiple mechanisms have been proposed, including microvascular obstruction, distal embolization, no-reflow phenomenon, and reperfusion injury [5]. Post-EVT CT perfusion (CTP) has emerged as a sensitive imaging biomarker for detecting tissue-level reperfusion failure, even when modified Thrombolysis in Cerebral Infarction (mTICI) scoring suggests complete reperfusion. [6] Persistent Tmax >6 s hypoperfusion despite TICI 3 grade has been documented in 20–50% of cases and is independently associated with worse functional outcome [5,7].

These observations have prompted increasing interest in adjunctive cerebroprotective strategies aimed at enhancing neurological recovery in patients with documented tissue-level reperfusion failure.

Cerebrolysin, a mixture of low-molecular-weight neuropeptides and free amino acids with established neurotrophic properties, includes attenuation of glutamate-mediated excitotoxicity, reduction of oxidative stress, modulation of post-reperfusion inflammatory cascades, and promotion of neuroplastic recovery. Clinically, it has demonstrated benefits in anterior circulation stroke when used alongside standard reperfusion therapy, with a favorable safety profile [8–13].

As part of an ongoing prospective study at the Department of Neurology, Shaare Zedek Medical Center (ClinicalTrials.gov Identifier: NCT06070753; institutional Helsinki Committee/IRB approval number SZMC-21-0347), eligible patients with anterior circulation large vessel occlusion who undergo mechanical thrombectomy (MT) receive adjunctive Cerebrolysin. Here, we report a representative case from this cohort demonstrating dissociation between post-EVT angiographic and tissue-level reperfusion, with favorable clinical recovery observed temporally following adjunctive Cerebrolysin therapy. This report was prepared in accordance with the CARE (CAse REport) reporting guidelines, and a completed CARE checklist is provided as a supplementary file.

Supplementary File

## Patient information

A 57-year-old single man with a history of long-standing severe smoking, peripheral vascular disease, and prior right superficial femoral artery stenting (2021), and with a remote history of substance abuse, presented with acute-onset left-sided weakness consistent with a wake-up stroke.

He was not taking any regular medications, including no antiplatelet or anticoagulant therapy. His baseline modified Rankin Scale (mRS) score was 0; he had been fully independent. There was no previous history of stroke, transient ischemic attack, or cognitive impairment. No relevant family history of cerebrovascular disease was reported.

## Clinical findings

The patient had gone to sleep at approximately 02:00 in his usual neurological state. He awoke at approximately 05:00 with sudden onset of left-sided weakness, was unable to rise from bed, and fell while attempting to stand, sustaining a left-sided contusion. He called emergency medical services and was admitted to the emergency department at 08:27, within the 24-hour last-known-well window applicable to wake-up stroke (Table 1).

**Table 1:** Clinical progression and treatment timeline

Day	Clinical course and key therapeutic interventions
Day 0	Wake-up stroke with left hemiparesis (NIHSS 7); last known well ~02:00, ED arrival 08:27. CT/CTA/CTP at 08:40 demonstrated tandem right ICA–M1 occlusion due to large-artery atherosclerosis. MT with carotid stenting (10:05–10:34) achieved TICI 3 recanalization; no intravenous thrombolysis was given. Post-procedural CTP at 11:16 revealed persistent residual hypoperfusion. Adjunctive Cerebrolysin therapy was initiated at 12:24.
Days 1–3	Continued adjunctive Cerebrolysin therapy. Sustained motor recovery and mild residual cognitive deficits noted.
Day 5	Discharged with NIHSS 1 on dual antiplatelet, antihypertensive, and lipid-lowering therapy; referred for community occupational therapy follow-up.

On admission, he was fully alert and oriented to person, place, and time. Vital signs were notable for a blood pressure of 190/93 mmHg, heart rate 80 beats per minute, and oxygen saturation 99% on room air. Neurological examination demonstrated:

- Moderate left-sided hemiparesis (left upper limb Medical Research Council grade 3/5; left lower limb 4/5)
- Left-sided sensory neglect
- Central facial palsy on the left
- Mild dysarthria
- Equal and reactive pupils with intact extraocular movements

The National Institutes of Health Stroke Scale (NIHSS) score on admission was 7.

## Diagnostic assessment

In accordance with the acute stroke treatment protocol of Shaare Zedek Medical Center for wake-up stroke presenting within 24 hours of last-known-well, the patient underwent non-contrast CT, CT angiography (CTA), and CT perfusion (CTP) at 08:40 to evaluate for large-vessel occlusion and salvageable penumbra. Non-contrast brain CT excluded intracranial hemorrhage. CTA demonstrated tandem occlusion involving the right internal carotid artery (RICA) and the M1 segment, with atherosclerotic plaque and total occlusion of the RICA at the carotid bifurcation, and poor distal filling. Plaque morphology — including the degree of stenosis proximal to the occlusion, surface characteristics, and the presence of ulceration, superimposed thrombus, or dissection — was not further characterized on CTA or DSA beyond the presence of atherosclerotic disease with total occlusion at the bifurcation.

Pre-procedural CTP (Figure 1A and B) demonstrated a large right anterior circulation perfusion deficit, with Tmax >6 s, volume of 74.73 cm^3^, an ischemic core (rCBF <30%) of 33.23 cm^3^, a penumbra of 41.5 cm^3^, and a mismatch ratio of 2.25. These findings indicated salvageable tissue and supported eligibility for MT.

**Figure 1:**
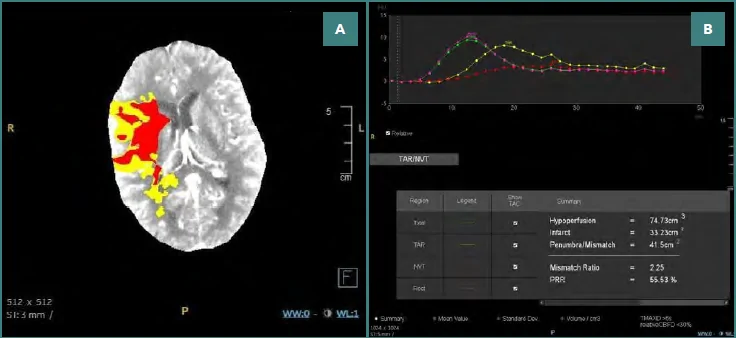
Pre-procedural computed tomography perfusion. A, Color overlay demonstrating a large area of estimated ischemic core (red, rCBF <30%) within the right anterior circulation territory, surrounded by penumbral hypoperfusion (yellow, Tmax >6 s). B, Automated quantitative summary showing a hypoperfusion volume of 74.7 cm^3^, an ischemic core of 33.2 cm^3^, a penumbral mismatch of 41.5 cm^3^, and a mismatch ratio of 2.25. rCBF, relative cerebral blood flow; Tmax, time to maximum of the residue function.

Digital subtraction angiography (Figures 2 and 3) confirmed the tandem occlusion, demonstrating severe atherosclerotic disease with total occlusion of the right internal carotid artery at the bifurcation. Following stent placement in the right ICA and MT, complete macrovascular recanalization (TICI grade 3) was achieved (Figure 3).

**Figure 2:**
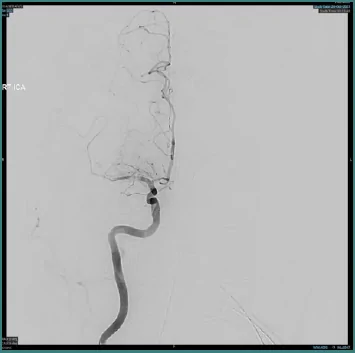
Pre-thrombectomy digital subtraction angiography (DSA) demonstrating the diseased right internal carotid artery, consistent with tandem RICA–M1 occlusion due to underlying large-artery atherosclerosis.

**Figure 3:**
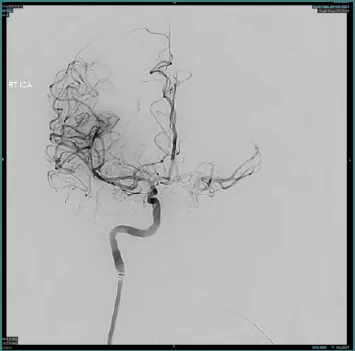
Post-thrombectomy digital subtraction angiography demonstrating complete recanalization (TICI grade 3) of the right anterior circulation following internal carotid artery stenting and MT. Robust filling of the right middle cerebral artery branches is now visible.

Despite complete angiographic recanalization, post-procedural CTP performed immediately (Figure 4) demonstrated persistent residual hypoperfusion. Scattered residual deficits were present in the right middle cerebral artery territory, indicating that complete angiographic reperfusion did not equate to complete tissue-level reperfusion (Figure 5). This finding identified the patient as a candidate for adjunctive cerebroprotective therapy.

**Figure 4:**
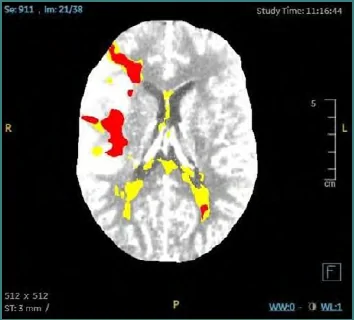
Post-procedural CTP color overlay performed immediately after MT. Despite TICI 3 recanalization, scattered residual ischemic core (red) and penumbra (yellow) persist within the right middle cerebral artery territory, consistent with microvascular reperfusion failure (no-reflow phenomenon).

**Figure 5:**
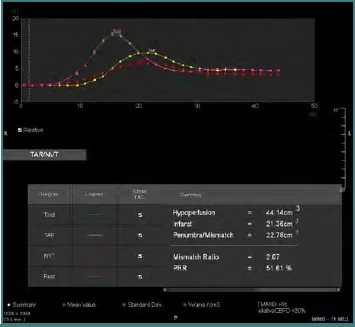
Post-procedural CTP automated quantitative summary. Hypoperfusion volume = 44.14 cm^3^, ischemic core = 21.36 cm^3^, penumbral mismatch = 22.78 cm^3^, mismatch ratio = 2.07. Although volumes are reduced compared with the pre-procedural study, significant tissue-level hypoperfusion persists despite TICI 3 angiographic recanalization.

Follow-up non-contrast brain CT (Figure 6) demonstrated evolution of acute ischemic changes within the right middle cerebral artery territory, without hemorrhagic transformation. All CT and CTP imaging was performed on a Siemens SOMATOM Force scanner (non-contrast CT acquisition parameters: 100 kV, 283 mA; CT perfusion source images: 5 mm slice thickness; derived color perfusion maps: 3 mm slice thickness), and CTP datasets were processed using Siemens Syngo.via software (version VB60A); no manual corrections (e.g., to the arterial input or venous output function) were required during post-processing. All images were reviewed by a senior neuroradiologist and judged to be of adequate diagnostic quality (raw imaging data and full acquisition/post-processing parameters available upon request).

**Figure 6:**
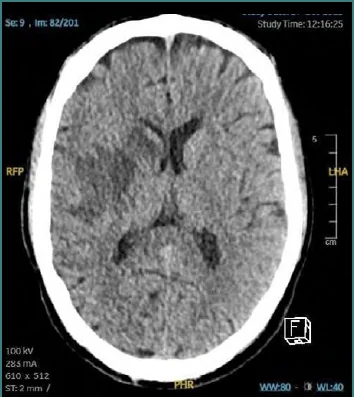
Follow-up non-contrast brain CT performed 24 hours after acute treatment

Laboratory evaluation revealed admission hyperglycemia; other parameters were within normal limits.

Stroke work-up, including transthoracic echocardiography with bubble study and 24-hour Holter monitoring, did not identify a cardioembolic source. Given the CTA and DSA findings of atherosclerotic plaque with total occlusion of the right ICA at the bifurcation, the stroke etiology was classified as large-artery atherosclerosis according to Trial of Org 10172 in Acute Stroke Treatment (TOAST) criteria. The tandem M1 occlusion was considered most consistent with artery-to-artery embolism from the diseased carotid bifurcation, a recognized mechanism of large-artery atherosclerotic stroke, rather than a separate embolic source.

## Therapeutic intervention

No intravenous thrombolysis was administered. The patient underwent MT with placement of a right internal carotid artery stent, performed from 10:05 to 10:34. Dual antiplatelet therapy (DAPT) was loaded peri-procedurally with 300 mg clopidogrel and 300 mg aspirin to maintain stent patency.

Post-procedural CTP, repeated at 11:16, revealed persistent residual hypoperfusion despite complete angiographic recanalization. Adjunctive Cerebrolysin (30 mL diluted in 250 mL saline, daily intravenously) was administered because of this documented perfusion deficit, with the first dose given at 12:24 the same day and treatment continued for four consecutive days.

Secondary prevention measures included continued dual antiplatelet therapy, low-dose ramipril for blood pressure control, and lipid-lowering therapy. The patient was assessed by physiotherapy and occupational therapy during the inpatient stay; no additional structured inpatient rehabilitation program was undertaken beyond these assessments.

## Follow-up and outcomes

Immediately following MT, the patient demonstrated marked neurological improvement, with the NIHSS score decreasing from 7 to 1, reflecting full motor and language recovery with only mild residual dysarthria. He was transferred to the neurological intensive care unit for monitoring.

Blood pressure decreased from 190/93 mmHg at presentation to 111/66 mmHg following the procedure, and the early hospital course was complicated by brief hemodynamic instability requiring transient noradrenaline support, followed by stabilization and initiation of low-dose ramipril for blood pressure control.

Over the subsequent days, motor function remained fully recovered. During the 5-day hospitalization, the patient was assessed by a physiotherapist, who found no functional motor deficit, and by an occupational therapist, who noted mild slowing of information processing. Outpatient follow-up with an occupational therapist in the community was therefore recommended.

No adverse effects related to Cerebrolysin therapy were observed during the inpatient stay. At discharge on hospital day 5 (31 October 2023), the patient had a discharge NIHSS score of 1, with mild residual cognitive impairment as the primary remaining concern. He was discharged on dual antiplatelet therapy, ramipril, and lipid-lowering therapy, and referred for outpatient occupational therapy follow-up in the community.

## Discussion

This case illustrates several critical aspects of contemporary stroke management and provides an important clinical example of the dissociation between angiographic and tissue-level reperfusion that can occur even after technically successful MT.

First, the patient’s presentation as a wake-up stroke with tandem right internal carotid artery and M1 segment occlusion represents a clinically challenging scenario requiring imaging-based selection for late-window MT [14]. A significant pre-procedural ischemic core (33 cm^3^) with a favorable mismatch ratio (2.25) supported the decision to proceed with MT and carotid artery stenting.

Second, and most importantly for this case, the post-procedural CTP findings revealed a clinically significant disparity between the angiographic outcome (TICI grade 3, indicating complete macrovascular recanalization) and the actual tissue-level reperfusion (persistent Tmax >6 s volume of 44 cm^3^ and residual ischemic core of 21 cm^3^). This phenomenon — sometimes termed the “no-reflow phenomenon” or microvascular reperfusion failure — has been documented in 20–50% of patients with technically successful thrombectomy and is increasingly recognized as a critical mechanism underlying futile recanalization [4,5]. Recent systematic reviews have established that post-EVT CTP–defined residual hypoperfusion is independently and consistently associated with worse functional outcome, identifying patients who may benefit from adjunctive cerebroprotective intervention [5–7,15].

Third, despite the substantial residual perfusion deficit on post-procedural imaging, the patient demonstrated dramatic acute clinical improvement (NIHSS 7 to 1) following the procedure. While spontaneous reperfusion of penumbral tissue and early recovery of stunned but viable brain tissue likely contributed to this rapid improvement, the magnitude of clinical recovery despite substantial documented residual hypoperfusion raises the hypothesis-generating possibility that adjunctive Cerebrolysin contributed to cerebroprotection in this patient. However, because Cerebrolysin was initiated at the same time as this improvement was already unfolding, no temporal interval separates the effect of TICI 3 recanalization from any potential treatment effect of Cerebrolysin, and this association cannot be established from a single case; spontaneous recovery and reperfusion-related penumbral salvage remain equally plausible explanations.

Nevertheless, single-case observations cannot establish causality between adjunctive Cerebrolysin and the favorable clinical course. Spontaneous recovery and the contribution of successful reperfusion to penumbral salvage likely both contributed to the observed outcome. The persistence of mild cognitive deficits at discharge, despite full motor recovery, underscores the multidimensional nature of post-stroke recovery and highlights the importance of comprehensive cognitive assessment beyond standard motor and language scales. A further limitation of this report is that outcome assessment was limited to the 5-day inpatient stay; standardized longer-term functional and cognitive outcome measures (e.g., 90-day modified Rankin Scale, formal neuropsychological testing) were not available for this report.

This case also illustrates the value of routine post-procedural CTP as a clinical tool for both prognostication and patient selection for adjunctive therapy. Patients with documented residual tissue-level hypoperfusion despite TICI 3 recanalization represent a clinically and biologically distinct subgroup who may benefit from adjunctive cerebroprotective strategies in addition to standard reperfusion therapy.

Ongoing prospective studies, including those at our institution, are expected to further clarify the therapeutic role of adjunctive Cerebrolysin and other cerebroprotective agents in patients with persistent post-thrombectomy perfusion deficits [15].

## Conclusion

This case illustrates that angiographic success (TICI grade 3) after mechanical thrombectomy does not guarantee complete tissue-level reperfusion, and that routine post-procedural CT perfusion can reveal a clinically meaningful residual hypoperfusion deficit even when the endovascular procedure itself is considered fully successful. In this patient, adjunctive Cerebrolysin was administered in this context and was followed by marked early clinical improvement (NIHSS 7 to 1); however, because Cerebrolysin was started without a comparator and no temporal separation exists from the patient's spontaneous recovery, a causal contribution of Cerebrolysin cannot be established, and spontaneous recovery and reperfusion-related penumbral salvage remain equally plausible explanations. Post-thrombectomy CTP may nonetheless be a useful tool for identifying patients with residual tissue-level perfusion deficits who could be candidates for adjunctive cerebroprotective strategies, and prospective controlled studies — including our institution's ongoing trial (ClinicalTrials.gov NCT06070753) — are needed to determine whether CTP-guided patient selection for adjunctive therapy improves outcomes after otherwise successful endovascular treatment.

## Patient perspective

The patient reported satisfaction with the rapid recovery of motor function and the ability to return to independent living. He acknowledged ongoing mild cognitive challenges, particularly with memory and complex task planning, and expressed motivation to continue with outpatient cognitive rehabilitation.
