# Supplementary material for: Adjunctive Cerebrolysin therapy following mechanical thrombectomy for tandem right internal carotid artery and middle cerebral artery occlusion with residual post-procedural perfusion deficit: a case report
Source: J Med Life. 2026 Aug;19(8):675–80. doi: 10.25122/jml-2026-0102 (PMC13674049; doi:10.25122/jml-2026-0102)
Supplement: Supplementary file 1 [file JMedLife-19-675-s001.pdf]

Supplementary File. CARE Checklist - Cerebrolysin Case Report

Table 1. CARE Checklist (2013)

CARE Topic	Item	Status	Location in Manuscript
Title	The area of focus and 'case report' should be included in the title.	Reported	Title page — "...A Case Report"
Keywords	2 to 5 key words that identify diagnoses or interventions in this case report, including "case report."	Reported	KeyWords section
Abstract (Introduction)	What is unique about this case and what does it add to the literature?	Reported	Abstract
Abstract (Main symptoms/ findings)	The main symptoms/findings of the patient.	Reported	Abstract
Abstract (Diagnosis/ Intervention/Outcome)	The main diagnoses, interventions, and outcomes.	Reported	Abstract
Abstract (Conclusion)	What is the main "take-away" lesson from this case?	Reported	Abstract; Discussion
Introduction	One or two paragraphs summarizing why this case is unique, with reference to relevant medical literature.	Reported	Introduction
Patient Information	De-identified demographic and other patient-specific information.	Reported	Patient Information
Patient Information	Main concerns and symptoms.	Reported	Patient Information; Clinical Findings
Patient Information	Medical, family, and psychosocial history, including relevant genetic information; relevant past interventions and their outcomes.	Reported	Patient Information
Clinical Findings	Describe relevant physical examination (PE) findings.	Reported	Clinical Findings
Timeline	Historical and current information from this episode of care, organized as a timeline (figure or table).	Reported	Timeline (Table 1)
Diagnostic Assessment	Diagnostic testing methods (e.g., PE, laboratory, imaging, questionnaires); diagnostic challenges; diagnostic reasoning, including other diagnoses considered; prognostic characteristics where applicable.	Reported	Diagnostic Assessment
Therapeutic Intervention	Types of intervention (e.g., pharmacologic, surgical, procedural); administration of intervention (e.g., dosage, strength, duration); changes in intervention, with rationale.	Reported	Therapeutic Intervention
Follow-up and Outcomes	Clinician- and patient-assessed outcomes, if available; important follow-up diagnostic and other test results; intervention adherence and tolerability (how was this assessed?); adverse and unanticipated events.	Reported	Follow-up and Outcomes
Discussion	Strengths and limitations of the management of this case.	Reported	Discussion
Discussion	Discussion of the relevant medical literature.	Reported	Discussion; Introduction
Discussion	The rationale for conclusions, including an assessment of possible cause-and-effect.	Reported	Discussion (causal language regarding Cerebrolysin explicitly qualified)
Discussion	The primary "take-away" lesson from this case (without over-generalization).	Reported	Discussion, final paragraph
Patient Perspective	The patient should share their perspective or experience, when appropriate.	Reported	Patient Perspective
Informed Consent	Did the patient give informed consent? Please provide if requested.	Reported	Informed Consent

Reference: Gagnier JJ, Kienle G, Altman DG, Moher D, Sox H, Riley D; CARE Group. The CARE guidelines: consensus-based clinical case reporting guideline development. BMJ Case Rep. 2013.

© 2026 by the authors. This article is an open access article distributed under the terms and conditions of the Creative Commons Attribution (CC BY 4.0) license.
